# On Citing Dobzhansky about the Significance of Evolution to Biology

**DOI:** 10.1093/iob/obac047

**Published:** 2023-01-04

**Authors:** Stefano Giaimo

**Affiliations:** Department of Evolutionary Theory, Max Planck Institute for Evolutionary Biology, 24306 Plön, Germany

## Abstract

Evolutionary thinking illuminates biology. Dobzhansky advocated this view in two distinct papers. The earliest paper (1964) is a discussion of the relationship between distinct biological disciplines, and one of the key ideas is that evolution is an integrative principle of biology. The later paper (1973) is a long argument to the effect that evolution makes more sense of the living than some creationist doctrines. The first paper should then be the primary reference for those biologists who cite Dobzhansky to champion among their peers the added value of evolutionary thinking in a specific scientific problem. Here, looking at citation data, we find evidence that this expected referencing practice does not coincide with the actual referencing practice in the scientific literature.

## Introduction

The geneticist Theodosius G. Dobzhansky famously stated that “nothing in biology makes sense except in the light of evolution.” But, barring a minimal variation (“makes sense in biology” vs. “in biology makes sense”), the dictum is found in two separate papers of Dobzhansky (1964, p. 449; 1973, p. 125). As they address different readerships, have different argumentative structures and come to different conclusions, the two papers should not be cited interchangeably.

In the scientific literature, quotations of or allusions to Dobzhansky’s dictum are abundant, e.g. (Lahn et al. 2001; Ameisen 2004; Varki 2006; Avise and Ayala 2007; Valas et al. 2009; Haerty and Pontin 2014; Gerhart-Hines and Lazar 2015; Katz 2016; Seitz 2020). Arguably, biologists often invoke the dictum to champion among their peers the added value of evolutionary thinking in a specific scientific problem. Which of the two Dobzhansky’s papers where the dictum appears should be the primary reference in this case? One aim of the present work is to answer this question and, thus, establish a best practice of citation by reviewing Dobzhansky’s original articles. Special attention will be paid to the meaning attached to the dictum in each paper. The other aim of this work is to look at citation data about his two papers in the hope of getting some understanding of the actual citation practice in the scientific literature.

Before starting, it should be noted that there is no intention here to establish the unconditional supremacy of either paper. Both are deep in content, highly cited, and have had a profound cultural impact. A critical discussion of all topics these papers cover, however, would go well beyond the scope of the present work, which instead focuses on a much more specific issue: the accuracy of citing one or another paper when invoking Dobzhansky’s dictum to remind other scientists of the relevance of an evolutionary perspective on a specific problem of interest.

## Dobzhansky’s two papers

### The dictum in Dobzhansky (1964)

First of all, we briefly review the two papers by Dobzhansky (1964, 1973) with a focus on the meaning of the dictum in each. He presented the first paper at the 1964 meeting of the American Society of Zoologists (later renamed the Society for Integrative and Comparative Biology). There, he applauded the ever-growing success of molecular biology in elucidating the fine mechanisms of the living. In particular, he attributed this success to the perpetuation in molecular biology of Descartes’ idea that organisms could be studied just like machines by decomposing them into components without appealing to any form of vitalism, which roughly is the view that living and nonliving matter fundamentally differ (Nicholson 2013). At the same time, Dobzhansky warned his colleagues of being tempted to reduce all of biology to the molecular level or to the functioning of isolated components. Life displays “a hierarchy of levels of biological integration [...]: molecule, cellular organelle, cell, tissue, organ, individual, Mendelian population, species, community, ecosystem” (Dobzhansky 1964, p. 444). Molecular biology studies the bottom level, while organismic biology, as designated by Dobzhansky (1964, p. 445), is the integrated study of levels above the molecular. Levels are intertwined so that “elementary phenomena and regularities on each succeeding level are organized patterns of those on the preceding level” (Dobzhansky 1964, p. 447). In this sense, Dobzhansky admitted, organismic biology can be seen as the study of patterns, and patterns of patterns, of molecular phenomena. Yet, he added, relations between levels of biological organization are so complex that even advanced knowledge at one level may fail to inform a meaningful understanding of the upper level. As an example, Dobzhansky recalled how the biochemistry of DNA, a discipline then celebrated for its enormous progress, was still insufficient to infer in an obvious way the existence of genes and Mendelian inheritance.

Beside the problem of how to deal with the complexity inherent to the hierarchical structure of life, Dobzhansky saw another major limitation in a purely molecular perspective on biology. He argued that only evolutionary thinking, which Dobzhansky found more prevalent in organismic than in molecular biology, enables those who investigate specific biological mechanisms to step back and look at the big picture of life. In particular, the evolutionary principle of common ancestry can explain otherwise puzzling similarities, including molecular ones, among distant organisms, while the evolutionary principle of adaptation can explain the biodiversity of these organisms as due to selection molding them differently in different environments. The famous dictum in this paper then points to the remarkable ability of evolutionary thinking, as opposed to purely mechanistic thinking, to ultimately integrate distinct biological observations into a coherent view (Grant 2000, p. 9).

### The dictum in Dobzhansky (1973)

The final part of the 1964 paper is a discussion of the importance of biology to philosophical issues. An example made by Dobzhansky is the realization that humankind is a contingent product of the historical process of evolution. In a single line within this final discussion, one finds a reference to antievolutionists, who are rapidly dismissed by Dobzhansky as negligible in numbers, arguably among academic biologists. In this respect, his other paper (Dobzhansky 1973), the title of which coincides with the dictum, is markedly different. He presented it at the 1972 convention of the National Association of Biology Teachers in San Francisco, California. Following a period of virtual absence from the school curriculum because of religious opposition and legal controversies (Larson 2003), evolution had progressively made its way through most biology textbooks sold in the United States during the late 1960s and early 1970s (Larson 2003; Scott 2009). This sparked intense antievolutionist reactions (Scott 2009), which, in turn, stimulated academic evolutionists to write extensively for the general public between the mid-1970s and the late-1980s (Scott 1997).

This historical and cultural backdrop, of which Dobzhansky was very aware (Burian 2005, p. 110), helps situate this second paper of his. In it, he did not carve out a special niche for evolutionary thinking, as opposed to mechanism-focused thinking, within biology. The crux of Dobzhansky (1973) is the potential tension between the evolutionary view of life and some creationist doctrines (Burian 2005, Ch. 6; Griffiths 2009; Dilley 2013). The introduction of the paper is about the conflict between science and religion. Using an anecdote, Dobzhansky goes back to the problem at the center of Galileo’s affair: Are the geocentric model and the heliocentric model of the solar system to be taken equally seriously? That the sun rotates around the earth, as the former model says, is prescribed by texts that are holy to some, while the rotation of the earth around the sun is scientifically established. The models contradict each other. But, according to Dobzhansky, the conflict is only apparent. In his view, ancient religious writings are symbolic narratives that concern with the meaning of human life: It would be a mistake to read them as a coherent picture of how things work. For this, scientific models are to be trusted, because they are meticulously built with the aim of making sense of the world according to the best available evidence. Dobzhansky then shifts his attention from physics to evolutionary biology.

The reader is asked to consider some undisputed biological observations: the existence of fossils, the stunning diversity of life, the apparently puzzling similarities between distinct species, and the uneven distribution of biodiversity. According to Dobzhansky, believing in the separate creation of each species by fiat puts those who embrace certain religious doctrines in an uncomfortable situation for the following reasons. Fossils could be meant to trick people into thinking that creation was not instantaneous. Highly elaborated organismal designs could be evidence of a capricious creator. The same creator would both jokingly introduce unnecessary commonalities among living beings and, out of absentmindedness, occasionally fill some taxa with exceedingly many species. But all this, Dobzhansky argued, is nonsensical blasphemy that should be rejected to accept instead that evolution, as the dictum goes, is the only way of making sense of those biological observations. A tree of life connects all living beings. Its ramifications, some dead some alive, form the evolutionary history, they span from a remote past to the present and the process of adaptation is one trigger of their branching, which does not follow any obvious pattern.

In the 1973 article, Dobzhansky placed special emphasis on common ancestry as evidence for the existence of evolution, since adaptation to the environment would be expected both under selection and under separate creation of the species (Griffiths 2009, p. 13). This contrasts with some interpretations of the dictum in this article as “an open invitation to interpret all biological phenomena as resulting from the process of natural selection” (Speakman 2013, p. 304). For a discussion of this adaptationist interpretation of Dobzhansky (1973), see Griffiths (2009). The final section of the 1973 paper restates the initial idea that the apparent conflict between science and religion disappears by accepting that religious writings are not of factual nature, they rather speak to the spiritual part of us.

### One dictum, two meanings

In summary, Dobzhansky’s dictum in his 1964 paper encapsulates one of the many ideas therein; specifically, the idea that evolutionary thinking offers an integrative view on biology by making sense of observations, like phylogenetically conserved traits, that mechanistic thinking alone cannot explain. The dictum in Dobzhansky’s 1973 paper instead condenses the logic of his argument against some creationist doctrines. Deep homologies, the skewness of the taxonomic distribution, and the existence of the fossil record (among other things) all make sense in the light of evolution. By rejecting evolution in favor of separate creation of the species, these same observations become evidence of attitudes of the creator that are at odds, at best, with traditional religious beliefs—but see Dilley (2013) for a discussion of the accuracy of Dobzhansky’s theological ideas.

After this review of Dobzhansky’s papers, we can go back to our initial issue. It is well established that evolution is one of the fundamental, integrative principles within biology (Smocovitis 1992; Pears 2003; Wake 2003; Danos et al. 2022). In this respect, Dobzhansky’s 1964 paper would appear to be more appropriate a reference than his 1973 paper for those scientists who want to champion among their peers, who are presumably already persuaded of the reality of evolution, of how valuable an evolutionary perspective can be in broadening one’s horizon on a given scientific problem. For this specific purpose, Dobzhansky’s 1973 paper instead seems not to be directly relevant. This second paper can be understood as his effort to equip teachers, especially those at the K–12 level, with arguments to address doubters of evolution and, thus, contrast any infiltration of creationist doctrines within the classroom during biology hours. No doubt that, when Dobzhansky’s dictum is quoted or alluded to by authors, their intended meaning may be entirely apparent from their usage of it regardless of the exact paper by Dobzhansky that is cited. However, referencing one or the other paper brings with it a whole different background.

## Understanding the current practice

What is the actual citation practice among scientists, in general, and among biologists, in particular, who invoke Dobzhansky’s dictum to remind their colleagues of how useful an evolutionary perspective on a given issue may be? A direct, accurate answer to this question would require a qualitative assessment of all academic publications in biology that report the dictum to understand its usage in each case. However, such endeavor would immediately face complications. First of all, a quick Google Scholar query reveals that publications containing either the string “nothing in biology makes sense” or the string “nothing makes sense in biology” are in the thousands, which makes a case-by-case approach unfeasible. It also remains unclear which of the returned citations should be categorized as coming from research in biology. Moreover, due to the catch-all strategy of the search engine, these citations are likely to be of different scholarly quality.

### Filtered citation data

To overcome the mentioned complications, we tried to answer our initial question indirectly. We reasoned that authors who invoke Dobzhansky’s dictum would cite either his 1964 article or his 1973 article. We then obtained vetted citations during the period 1964–2021 to these two articles by querying two databases, Scopus and BIOSIS Citation Index (Web of Science). Scopus is a multidisciplinary database, while BIOSIS Citation Index provides specialized coverage of biology and related disciplines. In our query to Scopus, we asked the database to exclude citations that it categorized as coming from the arts, the humanities, and the social sciences. Scopus returned 103 entries for Dobzhansky (1964) and 956 entries for Dobzhansky (1973). BIOSIS Citation Index returned 96 entries for Dobzhansky (1964) and 568 entries for Dobzhansky (1973).

Some citations to either article of Dobzhansky may correspond to instances where his dictum is invoked, but some may not. For example, Dobzhansky (1964) could be cited only in connection with the idea that biological systems are nested in a complex hierarchy of levels of organization without any mention to the dictum. But, as seen in the previous section, Dobzhansky (1973) is entirely centered about the relationship between science and religion, in general, and between evolutionary biology and creationism, in particular. Of all citations obtained from Scopus and BIOSIS Citation Index, we then only kept those where title, abstract, or keywords did not contain terms, like “intelligent design” or “theology”—see Supplementary Information (Giaimo 2022) for details, that could be indicative of philosophical, historical or educational discussions about the relationship between science and religion. Note that full text is not among the item attributes included in these databases and, therefore, it could not be searched for the presence of these terms. The aim of this filtering was to retain as much as possible only citations from papers likely to be exclusively concerned with specific scientific problems. Based on our reading of Dobzhansky’s papers, the naive expectation about the selected citations was that those that refer to Dobzhansky (1964) would be more than those that refer to Dobzhansky (1973). This is because the 1964 article contains the famous dictum as well as a discussion of a number of other ideas about the structure and nature of biology, while the 1973 article, which has a specific focus on creationism, should be cited very rarely, if ever, by papers that are selected for their not touching upon this topic or related ones.

**Fig. 1:**
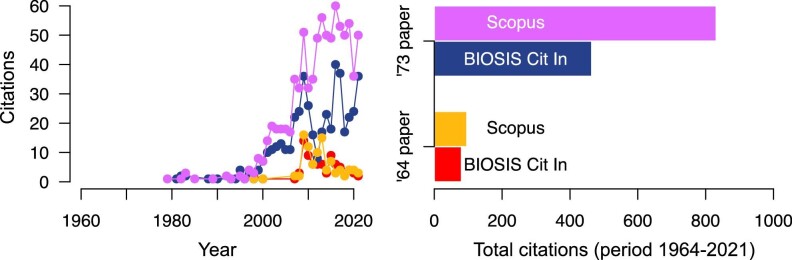
Citations to two papers by Dobzhansky on the significance of evolution. In discussing the relationship between biological disciplines, Dobzhansky (1964) reminded biologists of evolution as an integrative principle of their research field. Writing during a period of resurgence of creationism, Dobzhansky (1973) aimed at equipping biology teachers with the argument that evolution makes more sense of the living than the separate creation of species. Scopus is a multidisciplinary citation database. BIOSIS Citation Index is a citation database for biology and related disciplines. Citations to Dobzhansky’s papers in this figure (red and gold for the 1964 paper, blue and purple for the 1973 paper) are from scientific articles selected not to contain discussions about the relationship between science and religion. Color coding is consistent between the two plots. Supplementary Information (Giaimo 2022) details data retrieval, filtering, and visualization.

Filtered data (Fig. 1), however, were at variance with this naive expectation. Most citations to either paper of Dobzhansky were made in the last 20 years. Contrary to what expected, Dobzhansky’s 1973 paper has attracted many more citations than his 1964 paper. The gap is less marked when looking at citations from the life sciences as opposed to citations from all scientific disciplines. In both cases, however, the gap is wide.

A possible explanation for the discrepancy between the naive expectation and the data is that the expectation is too naive in assuming that citations are made strictly based on article content. In this respect, we should recall two factors. First, Dobzhansky’s dictum is so famous that most biologists may have come across it not by directly reading the original articles by Dobzhansky but via secondary sources (e.g., textbooks, articles, or scientific talks by other authors that mention the dictum). For example, the Australian and New Zealand Edition of *Campbell Biology* (Urri 2021), one of the leading introductory textbooks to general biology, reports (p. 11) the dictum in the form that is found in the 1973 paper. Second, Dobzhansky’s dictum appears as the title of his 1973 paper, while it is hidden in the text of the 1964 paper. These combined factors make of the 1973 article a much easier target for those authors looking for a reference to add to their manuscript as they quote a dictum they possibly already know from memory. This could be a reason that the 1973 article is disproportionally cited, compared to the 1964 article, by scientific works that should not discuss the relationship between science and religion. The prevailing use of online technologies such as citation databases and search engines in the last two decades, where most citations to either paper of Dobzhansky are concentrated, may have played a role too, as titles tend to be more visible than other publication attributes (e.g. full text) to some of these technologies.

### Most cited items

**Fig. 2:**
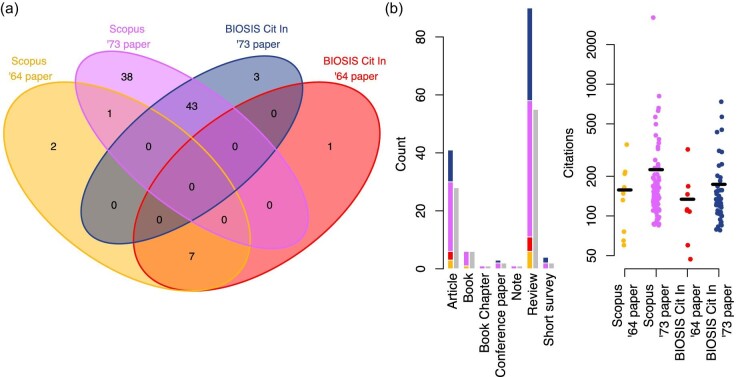
Summary statistics of the top-10% most-cited bibliographic items citing either Dobzhansky (1964) or Dobzhansky (1973). Items were among those retrieved for Fig. 1. **(a**) Venn diagrams showing unique and repeated items. **(b**) Histogram showing counts of different types of bibliographic items with (in color) and without (gray) repetitions and strip chart reporting (on log scale) the citations received by each item with horizontal black bars indicating averages. Color coding is consistent between the two panels. Supplementary Information (Giaimo 2022) details data retrieval and visualization.

The above explanation for the discrepancy between the naive expectation and citation data has a problematic aspect: It presumes that citations to Dobzhansky’s articles are typically done by authors who invoke his dictum. But, as noted before, references to his articles may be made for different purposes. To overcome this difficulty, we qualitatively looked at the top-10% most-cited bibliographic items in our filtered data. Citation numbers for these items were those reported among their attributes in the databases when the items were retrieved. The rationale for our citation-based selection, as opposed to random sampling, is that we deemed more cited items to be more likely to spread further their contained ideas and practices, including citation practices. This could enable us to capture, at least in part, any potential role of influential secondary sources in the prevalence of the 1973 paper within scientific citations. Figure 2 reports summary statistics of this sample. Most items found in BIOSIS Citation Index were also included in the more general Scopus database. Only a single item cited both Dobzhansky (1964) and Dobzhansky (1973). Reviews were the most prevalent type of bibliographic item. This is in line with scientometric research showing that reviews are usually more cited that research articles (Miranda and Garcia-Carpintero 2018). The top-10% most-cited items citing Dobzhansky (1973) received, on average, more citations than the top-10% most-cited items citing Dobzhansky (1964) both within and across databases.

The qualitative assessment of the top-10% most-cited items consisted in answering the following four questions for each item the full text of which was accessible:

- Q1: Does the item discuss or touch upon the topic of creationism?
- Q2: Is Dobzhansky’s dictum quoted or alluded?
- Q3: Is the item concerned with a scientific problem?
- Q4: Is the reference to Dobzhansky employed to introduce or justify an evolutionary perspective on this problem?

**Fig. 3:**
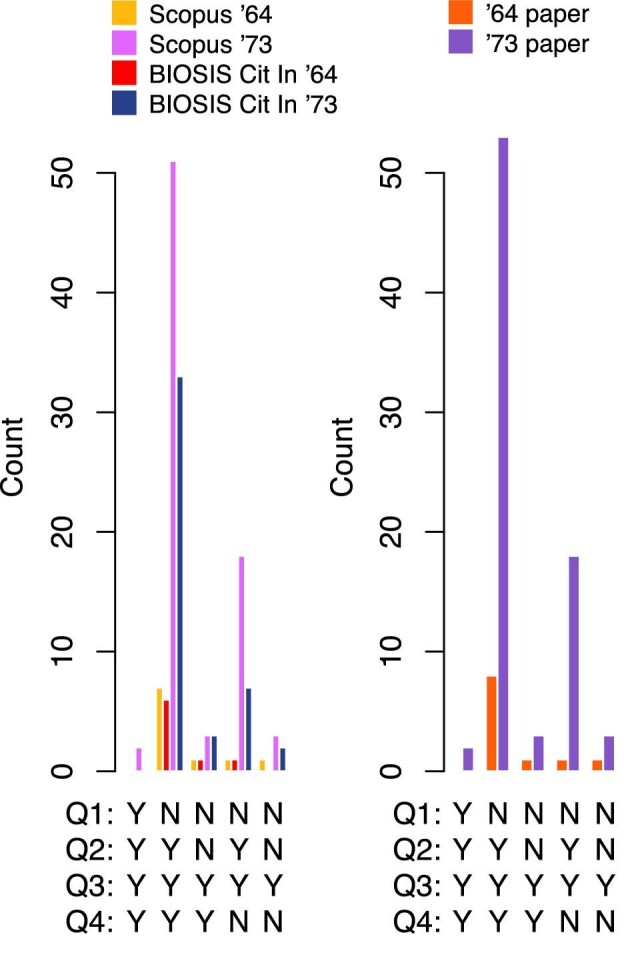
Qualitative analysis of the top-10% most-cited bibliographic items that cite either Dobzhansky (1964) or Dobzhansky (1973) and are selected not to contain discussions about creationism. Histograms report counts of different combinations of yes-or-no answer to Questions 1–4 (see main text) for each item. Only combinations that appeared at least once are reported. The histogram on the left reports results based on Dobzhansky’s cited paper and database, and the histogram on the right reports results only based on Dobzhansky’s cited paper independently of database. Supplementary Information (Giaimo 2022) details data retrieval and visualization.

Only for the single item citing both Dobzhansky (1964) and Dobzhansky (1973), these questions were answered twice, once with regard to the usage (especially, Q2 and Q4) of the reference to Dobzhansky (1964) and once with regard to the usage of the reference to Dobzhansky (1973). Answers to the four questions were in the form of “yes” (Y) or “no” (N). Thus, there were 16 possible combinations of different answers. Figure 3 reports counts of those combinations that appeared at least once. The very scarce presence of papers mentioning creationism (Q1: N) and the exclusive focus on specific scientific problems (Q3: Y) in these data are evidence of the effectiveness of our strategy to retrieve bibliographic items with precisely these two features. The most frequent combination of answers (NYYY) indicates that Dobzhansky’s two articles are typically referenced by scientists to quote his dictum as a reminder of the importance of evolutionary thinking in a specific scientific problem. Due to small sample size, the evidence is less strong in connection to the 1964 article, but a smaller sample size here reflects the smaller pool of citations gathered by this article compared to the 1973 article. The analysis also shows that a minor tendency exists to quote the dictum in Dobzhansky (1973) without associating it with the relevance of evolution or to a discussion of creationism, as evidenced by the count for the NYYN combination. Part of this tendency is due to papers that rephrase the dictum to give to it a new meaning, for example, “[j]ust as in biology ‘nothing makes sense except in the light of evolution’[...], the spontaneous changes that take place in macroscopic physicochemical systems cannot be understood except in the light of the second law of thermodynamics” (Frenkel 2015, p. 10) or “[t]hus with only a qualitative view of interactions, in reference to Dobzhansky [...], nothing in the biomolecular interaction network would make sense except in light of molecular complexes and the functional connections between them” (Bader and Hogue 2003, p. 25).

Extrapolating from these results and looking at the data in Figs. 1–2 suggest that the current referencing practice in biology generally is to preferentially cite Dobzhansky (1973), instead of Dobzhansky (1964), when his famous dictum is quoted to remind other biologists of the relevance of evolution. The connection of the 1973 paper with the issue of creationism appears to be scarcely appreciated. Hence, it is possible that many of the references to Dobzhansky (1973) do not derive from reading the original article—see also Griffiths (2009) on this—but from an easier access to it, as compared to the 1964 article, by authors looking for a citation to accompany Dobzhansky’s dictum. Since most-cited bibliographic items referencing Dobzhansky (1973) tend to be more cited than the most-cited items referencing Dobzhansky (1964), these data also indicate that there is a chance for this referencing practice to perpetuate and spread further if other authors get to know the dictum via secondary sources.

## Conclusions

It would seem that academic biologists who invoke Dobzhansky’s famous dictum to champion among their peers the adoption of an evolutionary perspective on a scientific problem should preferentially cite his 1964 paper over his 1973 paper. This is because the former paper, which was mostly directed at academic biologists, is about the relationships between biological disciplines and argues, among other things, for the role of evolution as an integrative principle of the field. In Dobzhansky’s view, evolution should complement a mechanism-focused approach in biology that alone cannot explain fundamental features of life like biodiversity and phylogenetically shared traits. In particular, evolution can make sense both of the diversity of living beings by appealing to their adaptation at different conditions and of their similarities by appealing to common ancestry. The 1973 paper, which was mostly directed at K–12 biology teachers, instead consists of a long argument against some creationist doctrines that, especially at the time when the paper was written, had a resurgence and tried to infiltrate school curricula. The logic of the argument is that evolution makes much more sense of the biological world than these doctrines. For example, Dobzhansky mentions the existence of fossils and homologies as facts easily accommodated by evolution, yet evidence of divine deceit if species had been created separately. References to the 1973 paper should then more naturally pertain to discussions about science education and the relationship between science and religion.

The current referencing practice prevailing in the scientific literature, however, seems different from expected. When academic biologists invoke Dobzhansky’s dictum to remind their colleagues of how relevant evolution may be to widen one’s horizon on a specific scientific problem, they appear to preferentially cite Dobzhansky (1973), and not Dobzhansky (1964). Clearly, when Dobzhansky’s dictum is quoted or alluded to in a scientific publication, the original context of the cited paper by Dobzhansky may not interfere with the message that the authors intend to convey by invoking the dictum. But since citations represent a kind of scientific currency both for authors and for journals (Penders 2018), correcting the current referencing practice appears important to ensure that credit is given where it is due.

## Competing interests

The author declares no competing interests.

## Data Availability Statement

All data and code are in the Supplementary Information for this submission. Supplementary Information is available in Giaimo (2022) (link: https://doi.org/10.6084/m9.figshare.21425604.v1).

## Author contributions statement

S.G. conceived the project, retrieved, and analyzed data and wrote the manuscript.

## Supplementary Material

obac047_Supplemental_Files
