# Supplementary figures and images for: On Citing Dobzhansky about the Significance of Evolution to Biology
Source: Integr Org Biol. 2023 Jan 4;5(1):obac047. doi: 10.1093/iob/obac047 (PMC9830543; doi:10.1093/iob/obac047)

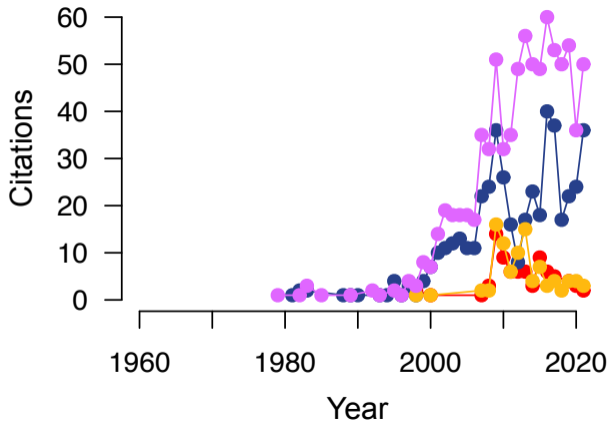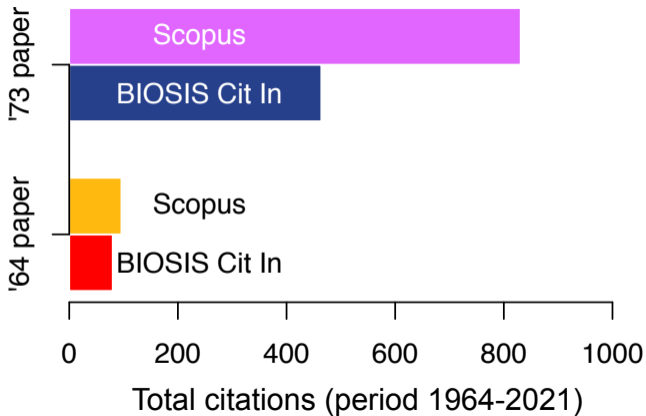

Supplement: obac047_Supplemental_Files [file obac047_supplemental_files.zip › fig1.pdf]

a)

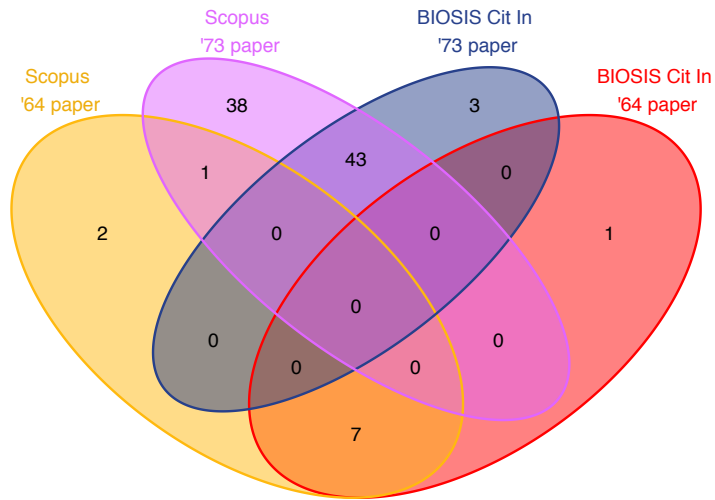

b)

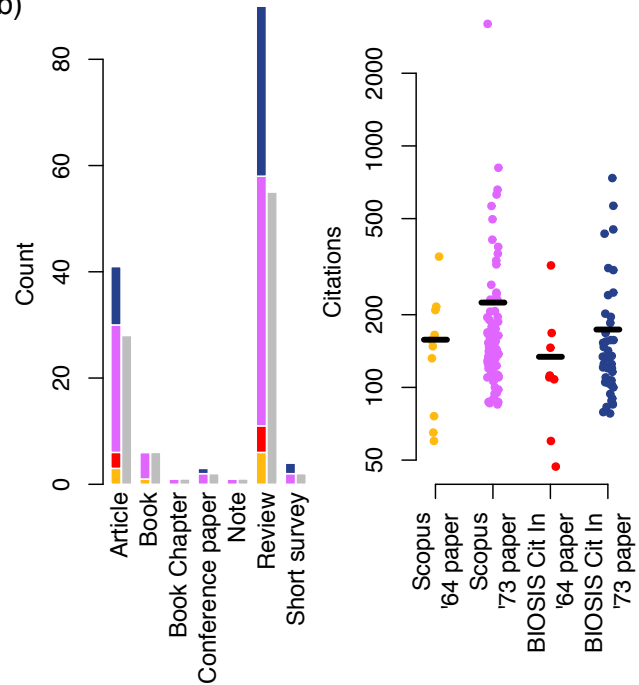

Supplement: obac047_Supplemental_Files [file obac047_supplemental_files.zip › fig2.pdf]

■ Scopus '64  
■ Scopus '73  
■ BIOSIS Cit In '64  
■ BIOSIS Cit In '73

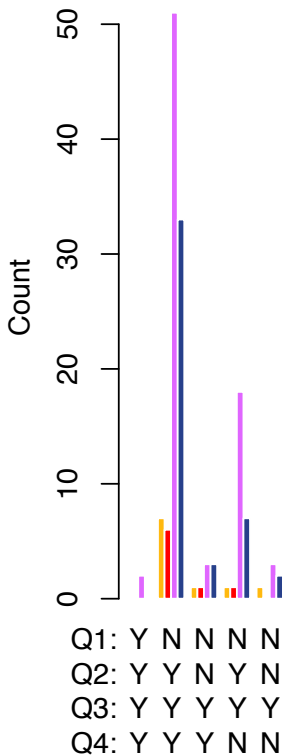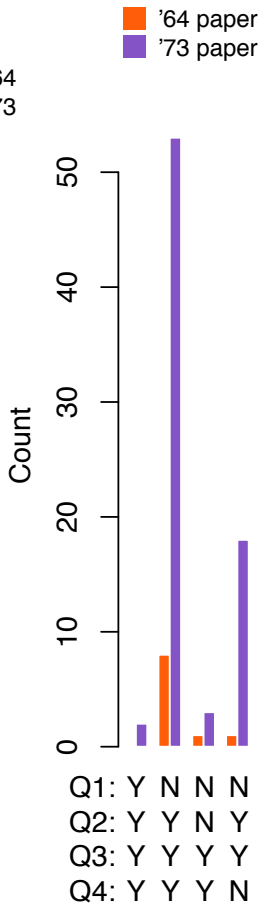

Supplement: obac047_Supplemental_Files [file obac047_supplemental_files.zip › fig3.pdf]
